# ICP0 Antagonizes ICP4-Dependent Silencing of the Herpes Simplex Virus *ICP0* Gene

**DOI:** 10.1371/journal.pone.0008837

**Published:** 2010-01-21

**Authors:** Mingyu Liu, Brandon Rakowski, Edward Gershburg, Carla M. Weisend, Olivier Lucas, Edward E. Schmidt, William P. Halford

**Affiliations:** 1 Department of Microbiology and Immunology, Southern Illinois University School of Medicine, Springfield, Illinois, United States of America; 2 Department of Veterinary Molecular Biology, Montana State University, Bozeman, Montana, United States of America; Institute of Infectious Disease and Molecular Medicine, South Africa

## Abstract

ICP0 is a regulatory protein that plays a critical role in the replication-latency balance of herpes simplex virus (HSV). Absence of ICP0 renders HSV prone to establish quiescent infections, and thus cellular repressor(s) are believed to silence HSV mRNA synthesis when ICP0 fails to accumulate. To date, an ICP0-antagonized repressor has not been identified that restricts HSV mRNA synthesis by more than 2-fold. We report the unexpected discovery that HSV's major transcriptional regulator, ICP4, meets the criteria of a *bona fide* ICP0-antagonized repressor of viral mRNA synthesis. Our study began when we noted a repressive activity that restricted ICP0 mRNA synthesis by up to 30-fold in the absence of ICP0. When ICP0 accumulated, the repressor only restricted ICP0 mRNA synthesis by 3-fold. ICP4 proved to be necessary and sufficient to repress ICP0 mRNA synthesis, and did so in an ICP4-binding-site-dependent manner. ICP4 co-immunoprecipitated with FLAG-tagged ICP0; thus, a physical interaction likely explains how ICP0 antagonizes ICP4's capacity to silence the *ICP0* gene. These findings suggest that ICP0 mRNA synthesis is differentially regulated in HSV-infected cells by the virus-encoded repressor activity embedded in ICP4, and a virus-encoded antirepressor, ICP0. Bacteriophage λ relies on a similar repression-antirepression regulatory scheme to “decide” whether a given infection will be productive or silent. Therefore, our findings appear to add to the growing list of inexplicable similarities that point to a common evolutionary ancestry between the herpesviruses and tailed bacteriophage.

## Introduction

During productive replication, ∼75 proteins are synthesized from the herpes simplex virus (HSV) genome in a temporal cascade [1]. Virion protein 16 (VP16) in the tegument of HSV virions forms a complex with the cellular transcription factor Oct 1 to initiate a cascade of viral gene expression [2], [3]. Only five immediate-early (IE) genes are initially induced based on the presence of VP16-responsive elements in their promoters [4]. Viral IE proteins such as infected cell proteins 0 (ICP0) and 4 (ICP4) are believed to play a key role in activating viral mRNA synthesis, and thus promoting the synthesis of ∼70 early (E) and late (L) proteins that replicate and package HSV genomes into new virions.

ICP0 was first identified based on its capacity to transform HSV's major transcriptional regulator, ICP4, from a weak transcriptional activator to a potent activator of mRNA synthesis; specifically, combinations of ICP0 and ICP4 are 20-fold more potent at driving mRNA synthesis than either ICP0 or ICP4 alone [5], [6]. Functionally, synthesis of ICP0 causes HSV's equilibrium to abruptly tip towards productive replication, whereas absence of ICP0 produces the opposite effect. Synthesis of ICP0 is sufficient to trigger HSV reactivation in trigeminal ganglion neurons and other models of latent HSV infection [7], [8]. HSV *ICP0* ^−^ viruses replicate to nearly wild-type levels when cells are inoculated with more than 1 plaque-forming unit (pfu) of virus per cell. At multiplicities of infection (MOI) below 0.1 pfu per cell, the same *ICP0* ^−^ viruses establish quiescent infections in 99% of the cells they infect [9], [10]. Such observations suggest that ICP0 antagonizes a repressor of HSV replication, whose repressive capacity can be saturated with high numbers of HSV genomes [10].

Preston and Everett first articulated the concept of an ICP0-antagonized repressor of HSV gene expression [11], [12], [13]. Because ICP0 is an E3 ubiquitin ligase [14], it was suggested that a repressor might silence HSV mRNA synthesis in the absence of ICP0, whereas synthesis of ICP0 would result in ubiquitination and/or proteasomal destruction of the repressor, thus allowing HSV mRNA synthesis to proceed unhindered [15]. Since the advancement of this hypothesis, many laboratories have joined in the search for the unidentified repressor(s) of HSV gene expression. Individual cellular proteins that co-localize with ICP0 have been probed for their capacity to function as repressors of HSV replication such as PML and Sp100 [16], [17]. Suspected pathways of silencing HSV genomes, such as epigenetic silencing, have been explored to determine if HSV *ICP0* ^−^ null viruses are more sensitive to these gene silencing mechanisms [18], [19], [20], [21]. Based on such inquiries, the list of proteins that may function as ICP0-antagonized repressors of HSV gene expression continues to grow and currently includes PML [16], [17], [18], [22], Sp100 [23], cyclin D3 [24], IRF-3 and IRF-7 [25], centromeric proteins CENP-B and CENP-C [26], [27], HDAC1/2-CoREST-REST [18], DNA-dependent protein kinase [28], and class II histone deacetylases [21].

The PML protein is among the most carefully scrutinized of the potential ICP0-antagonized repressors of HSV replication. In support of the PML repressor hypothesis, ICP0 and HSV genomes co-localize with PML nuclear bodies in cells [29], [30], synthesis of ICP0 triggers the dispersal of PML nuclear bodies [29], and PML is strongly induced by interferon treatments that are known to impede HSV replication [31]. Weaknesses in the PML repressor hypothesis include the fact that overexpression of PML does not inhibit HSV gene expression and does not interfere with the replication of wild-type HSV or *ICP0* ^−^ null viruses [Ref. 32; unpublished data of W. Halford]. Although siRNA-knockdown of both PML and Sp100 results in an ∼40-fold increase in the replication of HSV *ICP0* ^−^ null viruses in human fibroblasts, this represents less than a 1% complementation of the >10,000-fold repression that occurs in these cells [17]. Finally, there is no direct evidence of a physical interaction between ICP0 and PML [33].

Like many laboratories, we too have been interested in understanding why failure to synthesize ICP0 results in limited mRNA transcription from the HSV genome. Rather than interrogate specific proteins for their capacity to repress viral mRNA synthesis, we chose to establish an experimental system that would allow us to rapidly monitor repression of a single HSV IE gene, the *ICP0* gene. To this end, an *ICP0^−^* virus was constructed that bore an ∼750 bp insertion of green fluorescent protein (GFP) coding sequence and a stop codon in exon 2 of the *ICP0* gene. The resulting virus, HSV-1 0^−^GFP, synthesized a 3.5 kb ICP0^−GFP^ mRNA and a truncated ICP0^−GFP^ peptide. Using the GFP fluorescent reporter as a screening tool, we probed for conditions that alleviated or exacerbated repression of the *ICP0^−GFP^* reporter gene in HSV-infected cells such as presence or absence of biologically active ICP0.

With the aid of these new reagents, we report the identification of a protein that satisfied four empirical criteria that should be expected of an ICP0-antagonized repressor of HSV mRNA transcription; specifically, the identified protein ***1.*** was required to observe repression of ICP0 mRNA synthesis in HSV-infected cells; ***2.*** was sufficient to repress ICP0 mRNA synthesis in the absence of ICP0; ***3.*** was unable to silence ICP0 mRNA synthesis when ICP0 accumulated; and ***4.*** physically interacted with ICP0.

We report the unexpected finding that the viral IE protein, ICP4, satisfied all of the criteria expected of a *bona fide* ICP0-antagonized repressor of HSV mRNA transcription. It is relevant to note that ICP4's capacity to function as a repressor of HSV IE mRNA transcription is well established, particularly in the context of the *ICP4* and *L/ST* genes [34], [35]. Evidence has been presented both for and against the hypothesis that ICP4 represses the *ICP0* gene in HSV-infected cells [36], [37]. However, the perceived importance of the hypothesis may be measured in terms of the attention it has received; 14 years have elapsed since the last study was published that considered ICP4's potential to repress the *ICP0* gene [37].

We present new evidence that corroborates earlier findings that ICP4's capacity to repress ICP0 mRNA synthesis is indeed modest *when ICP0 accumulates*. However, when ICP0 fails to accumulate, then ICP4 is capable of silencing the *ICP0* gene. We present functional evidence that ICP0 antagonizes ICP4-dependent repression of the *ICP0* gene. In addition, we present the first direct evidence that ICP0 and ICP4 physically interact in HSV-infected cells. Collectively, these data suggest an alternative view of HSV gene regulation, which is consistent with the biology of a virus that establishes latent infections: *In the absence of ICP0*, *ICP4 functions as an efficient repressor of HSV IE mRNA transcription*, *whereas accumulation of ICP4's binding partner*, *ICP0*, *converts ICP4 from a repressor to an activator of HSV mRNA synthesis* *[5]*, *[6]*. The findings that led us to these unexpected conclusions are presented below.

## Results

### Characterization of HSV-1 Viruses That Contain a GFP Reporter in ICP0 ^−^ or ICP0^+^ Genes

**Figure 1:**
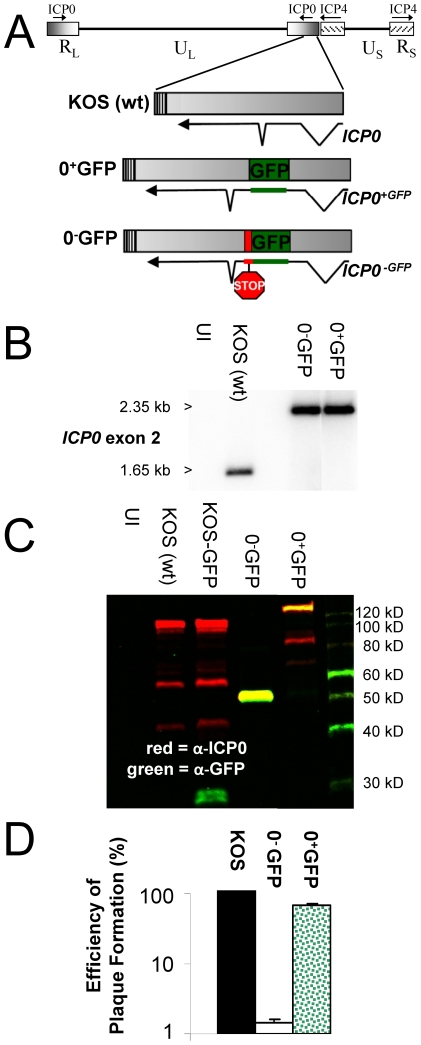
Characterization of HSV-1 0^−^GFP and 0^+^GFP. (A) Schematic and (B) Southern blot analysis of the wild-type *ICP0*, *ICP0^−GFP^*, and *ICP0^+GFP^* genes in HSV-1 KOS, 0^−^GFP, and 0^+^GFP, respectively. Southern blots show an oligonucleotide specific for intron 1 hybridized to a PshAI - BssHII fragment of the *ICP0* gene. (C) Two-color Western blot analysis of proteins harvested from cells that were uninfected (UI) or were inoculated with 5 pfu per cell of the HSV-1 KOS, KOS-GFP, 0^−^GFP, or 0^+^GFP viruses. ICP0 and GFP proteins were labeled with ICP0-specific monoclonal antibody 11060 (red bands) and rabbit polyclonal GFP-specific antibody (green bands). Yellow bands are those in which ICP0 and GFP were present in a single protein. (D) The percent efficiency of plaque formation cells in Vero cells (i.e., number of plaques per 100 pfu) of HSV-1 KOS, 0^+^GFP, and 0^−^GFP, as compared to the efficiency with which the same viral inocula formed plaques in ICP0-complementing L7 cells (n = 3 per group). As indicated on the logarithmic scale of the y-axis, HSV-1 0^+^GFP and 0^−^GFP formed plaques in Vero cells with 66±5% and 1.5±0.2% efficiency, respectively.

HSV-1 recombinant viruses carrying a *GFP* coding sequence in each copy of the *ICP0* gene were constructed for the purpose of studying how the *ICP0* gene is regulated. An *ICP0^+GFP^* gene was constructed that encoded an ICP0 protein that bore a GFP insertion between amino acids 104 and 105 (Fig. 1A). An ICP0-null control gene, the *ICP0^−GFP^* gene, encoded the N-terminal 104 amino acids of ICP0 fused to GFP (Fig. 1A). The chimeric *ICP0^+GFP^* or *ICP0^−GFP^* genes were introduced into the *LAT-ICP0* locus of HSV-1 strain KOS by homologous recombination to yield the HSV-1 viruses 0^+^GFP and 0^−^GFP, respectively (Fig. 1B; Table 1).

**Table 1:** Description of HSV-1 viruses.

Virus/Genotype	Phenotype/Properties	Reference
KOS (wild-type)	Wild-type laboratory strain of HSV-1 (12 passages removed from isolation).	[84]
n212 (*ICP0* ^−^ null)	Replicates with 1% efficiency at MOIs below 0.1 pfu per cell; virus synthesizes the first 211 of 775 amino acids of ICP0.	[43]
0^−^GFP (*ICP0* ^−^ null)	Replicates with 1% efficiency at MOIs below 0.1 pfu per cell; virus synthesizes the first 104 amino acids of ICP0 coupled to GFP.	this study
0^−^4BS^−^ (*ICP0* ^−^ null)	Replicates with 1% efficiency at MOIs below 0.1 pfu per cell; virus synthesizes the first 104 amino acids of ICP0 coupled to GFP. A 4-bp deletion disrupts the ICP4 DNA-binding site in the *ICP0* promoter.	this study
0^+^GFP (*ICP0* ^+^)	Replicates with 66% efficiency at MOIs below 0.1 pfu per cell; virus synthesizes full-length ICP0 with GFP inserted between amino acids 104 and 105.	this study
0^+^4BS^−^ (*ICP0* ^+^)	Replicates with 66% efficiency at MOIs below 0.1 pfu per cell; virus synthesizes full-length ICP0 with GFP inserted between amino acids 104 and 105. A 4-bp deletion disrupts the ICP4 DNA-binding site in the *ICP0* promoter.	this study
0^+^FLAG_24_ (*ICP0* ^+^)	Replicates with 100% efficiency at MOIs below 0.1 pfu per cell; virus synthesizes full-length ICP0 with FLAG epitope inserted between amino acids 23 and 24.	this study
RP4 (*VP16* ^−^ null)	Replicates with 1% efficiency at MOIs <0.1 pfu per cell; virus synthesizes VP16 protein lacking half of the acidic transactivation domain (Δa.a. 413–454 [of 490]).	[44]
n12 (*ICP4* ^−^ null)	Incapable of replication unless ICP4 is provided *in trans*; virus synthesizes the first 252 of 1298 amino acids of ICP4.	[50]
tsB21 (*ICP4* ^ts^)	Replication-competent at 34°; replication-defective at 39.5°C; virus synthesizes a temperature-sensitive ICP4 protein (lesion maps to the C-terminal third).	[47]
d27-1 (*ICP27* ^−^ null)	Incapable of replication unless ICP27 is provided *in trans*; virus bears a 1.6 kb deletion that removes 80% of the ICP27 ORF.	[48]
22/n199 (*ICP22* ^−^ null)	Virus replicates like wild-type in cell culture but fails to replicate in animals; virus synthesizes the first 198 of 420 amino acids of ICP22.	[49]

The ICP0^−GFP^ or ICP0^+GFP^ reporter proteins encoded by HSV-1 0^+^GFP and 0^−^GFP were analyzed by two-color Western blot analysis. In cells infected with wild-type HSV-1 KOS or KOS-GFP, ICP0-specific monoclonal antibody 11060 labeled the 110 kDa protein species that is ICP0, as well as two protein species of ∼40 and 55 kDa (red bands, Fig. 1C). In addition, the GFP-expressing recombinant virus, KOS-GFP [38], encoded an ∼30 kDa GFP protein that was labeled by rabbit anti-GFP antibody (green band in Fig. 1C). As expected, HSV-1 0^−^GFP encoded a single ∼55 kDa protein that was labeled by ICP0- *and* GFP-specific antibodies, while 0^+^GFP encoded an 140 kDa protein that was labeled with both ICP0- and GFP-specific antibodies (yellow bands in Fig. 1C).

The functionality of the ICP0^+GFP^ protein was compared to wild-type ICP0. Wild-type HSV-1 strain KOS formed plaques with 100% efficiency in Vero cells relative to the number of plaques that formed in ICP0-complementing L7 cells (Fig. 1D). In contrast, only ∼1% of HSV-1 0^−^GFP (*ICP0* ^−^) viruses formed plaques in Vero cells (Fig. 1D). HSV-1 0^+^GFP formed plaques in Vero cells at 66% efficiency relative to the number of plaques that formed in ICP0-complementing L7 cells (Fig. 1D). Thus, the ICP0^+GFP^ protein retained sufficient activity to promote HSV-1 0^+^GFP plaque formation with 45-fold greater efficiency than its matching *ICP0* ^−^ null control virus, HSV-1 0^−^GFP.

### Inefficient ICP0 ^−GFP^ mRNA Synthesis from HSV-1 0^−^GFP in the Absence of ICP0

ICP0 has been described as a promiscuous transcriptional activator [11], [19], [39]. We reasoned that ICP0 should act in a positive-feedback loop to promote mRNA synthesis from its own gene, the *ICP0* gene. To test this inference, an ICP0-expressing adenovirus vector, Ad-ICP0, was compared to a control vector, Ad-n212 (Table 2), for its capacity to stimulate the expression of the *ICP0^−GFP^* reporter gene embedded in the native *LAT-ICP0* locus of HSV-1 0^−^GFP.

**Table 2:** Description of ΔE1–E3 adenovirus vectors.

Virus	Promoter/Gene product	Reference
Ad-null	TRE promoter/no gene product	[7]
Ad-ICP0	TRE promoter/wild-type ICP0 (775 amino acids)	[7]
Ad-n212	TRE promoter/N-terminus of ICP0 (211 of 775 amino acids)	[7]
Ad-ICP4	TRE promoter/wild-type ICP4 (1298 amino acids)	[7]
Ad.CMV-TetOn	CMV promoter/TetOn (rtTA) protein	[7]

**Figure 2:**
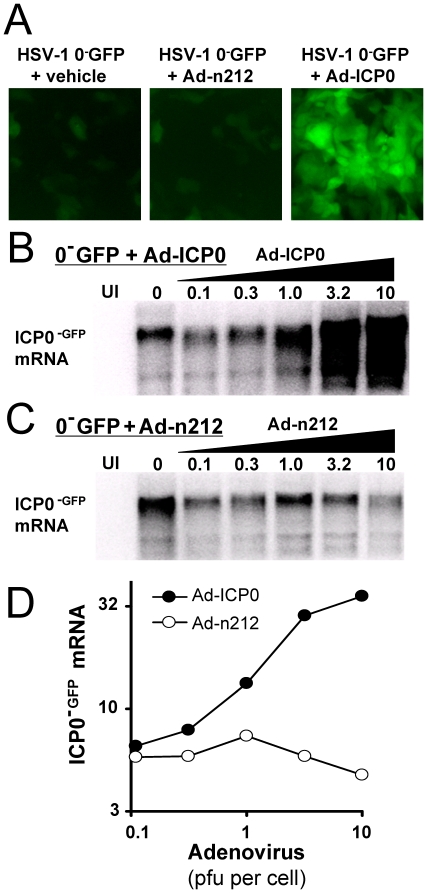
ICP0 stimulates HSV *ICP0^−GFP^* gene expression. (A) Photomicrographs of ICP0^−GFP^ protein fluorescence in Vero cells inoculated with 2.5 pfu per cell of HSV-1 0^−^GFP at 12 hours p.i., which were pre-treated with either vehicle or 10 pfu per cell of Ad-n212 or Ad-ICP0 for 12 hours (20× magnification). The TRE promoters in Ad-n212 and Ad-ICP0 were induced with 20 pfu per cell of Ad.CMV-TetOn and 10 µM doxycycline. (B and C) Northern blot comparison of ICP0^−GFP^ mRNA levels in cells that were uninfected (UI) or were inoculated with 2.5 pfu per cell of HSV-1 0^−^GFP and 0 to 10 pfu per cell of (B) Ad-ICP0 or (C) Ad-n212 (10 µg RNA per lane; RNA harvested at 12 hours p.i.). (D) ICP0^−GFP^ mRNA levels plotted as a function of the MOI of adenovirus in the pre-treatment.

In an initial test, cells were pre-treated with vehicle, Ad-n212, or Ad-ICP0, and were inoculated 12 hours later with 2.5 pfu per cell of HSV-1 0^−^GFP. When compared at 12 hours post-inoculation (p.i.), HSV-1 0^−^GFP expressed barely detectable levels of ICP0^−GFP^ fluorescence in cells pre-treated with vehicle or Ad-n212 (Fig. 2A). In contrast, pre-treatment with Ad-ICP0 allowed HSV-1 0^−^GFP to express high levels of ICP0^−GFP^ fluorescence (Fig. 2A).

Northern blot analysis was conducted to determine if adenovirus-encoded ICP0 stimulated ICP0^−GFP^ mRNA accumulation in HSV-1 0^−^GFP-infected cells. Vero cells were inoculated with 0 to 10 pfu per cell of Ad-ICP0, and were inoculated 12 hours later with 2.5 pfu per cell of HSV-1 0^−^GFP. Northern blot analysis with a GFP-specific probe demonstrated that Ad-ICP0 stimulated a 5-fold increase in ICP0^−GFP^ mRNA levels and did so in a dose-dependent manner (Fig. 2B, D). In contrast, Ad-n212 had no effect on ICP0^−GFP^ mRNA levels (Fig. 2C, D). Therefore, exogenous ICP0 stimulated the accumulation of ICP0^−GFP^ mRNA in cells inoculated with HSV-1 0^−^GFP.

### Efficient ICP0^−GFP^ mRNA Synthesis from HSV-1 0^−^GFP when Protein Synthesis Is Blocked

**Figure 3:**
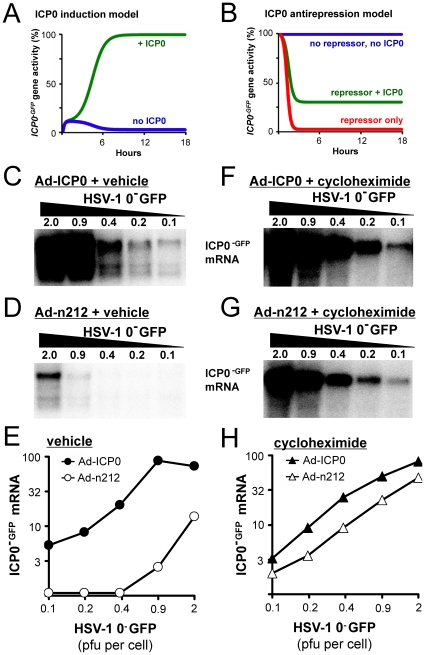
ICP0 de-represses HSV *ICP0^−GFP^* gene expression. (A and B) Alternative models by which ICP0 may stimulate *ICP0^−GFP^* gene activity. (A) An ICP0 induction model predicts that ICP0 will be required to induce the *ICP0^−GFP^* gene to its full activity (green line), whereas efficient *ICP0^−GFP^* gene expression will not occur in the absence of ICP0 (blue line). (B) An ICP0 antirepression model predicts that ***i.*** a repressor will silence the *ICP0^−GFP^* gene when ICP0 fails to accumulate (red line), *ii.* ICP0 prevents the repressor from silencing the *ICP0^−GFP^* gene (green line), and *iii.* ICP0 is not required for efficient *ICP0^−GFP^* gene expression when the repressor is absent (blue line). (C, D, F, G) Northern blots of ICP0^−GFP^ mRNA isolated from Vero cells inoculated with MOIs of 0.1 to 2 pfu per cell of HSV-1 0^−^GFP (RNA harvested 12 hours p.i.; 10 µg per lane). Vero cells were pre-treated with 10 pfu per cell of (C, F) Ad-ICP0 or (D, G) Ad-n212 whose TRE promoters were induced with 20 pfu per cell of Ad.CMV-TetOn and 10 µM doxycycline. Cells were treated with (C, D) vehicle or (F, G) 200 µM cycloheximide at the time of HSV-1 0^−^GFP inoculation. (E, H) ICP0^−GFP^ mRNA levels are plotted as a function of the MOI of HSV-1 0^−^GFP used to inoculate Vero cells treated with (E) vehicle or (H) cycloheximide.

We were interested to determine how ICP0 influences the activity of the *ICP0^−GFP^* gene in cells inoculated with HSV-1 0^−^GFP. One possibility was that ICP0 induced mRNA synthesis from all of HSV-1 0^−^GFP's genes, including the *ICP0^−GFP^* gene (Fig. 3A). A second possibility was that absence of ICP0 allowed a repressor such as PML [17] or HDAC1/2-CoREST-REST [19] to silence all of HSV-1 0^−^GFP's genes including the *ICP0^−GFP^* gene (Fig. 3B, red line). Under this latter hypothesis, ICP0 might stimulate *ICP0^−GFP^* mRNA synthesis by antagonizing the repressor of the *ICP0^−GFP^* gene (Fig. 3B, green line). An experiment was conducted to evaluate the relative likelihood of these two possibilities.

The effect of Ad-ICP0 versus Ad-n212 was compared in cells inoculated with MOIs of 0.1 to 2.0 pfu per cell of HSV-1 0^−^GFP. As expected, HSV-1 0^−^GFP expressed ICP0^−GFP^ mRNA at all MOIs so long as ICP0 was provided *in trans* from Ad-ICP0 (Fig. 3C). In cells treated with the Ad-n212 control vector, HSV-1 0^−^GFP failed to synthesize detectable levels of ICP0^−GFP^ mRNA at MOIs below 0.9 pfu per cell (Fig. 3D). At an MOI of 0.9 pfu per cell of HSV-1 0^−^GFP, 34-fold higher levels of ICP0^−GFP^ mRNA were noted in cells treated with Ad-ICP0 relative to cells treated with Ad-n212 (Fig. 3E). Thus, efficient ICP0^−GFP^ mRNA accumulation was highly dependent on ICP0 at low multiplicities of HSV infection.

The mechanism by which ICP0 promoted ICP0^−GFP^ mRNA accumulation was unclear. Two possibilities included ICP0-dependent induction of ICP0^−GFP^ mRNA synthesis (Fig. 3A) or ICP0-dependent antirepression of ICP0^−GFP^ mRNA synthesis (Fig. 3B). To differentiate between these possibilities, cells were inoculated with Ad-ICP0 or Ad-n212 for twelve hours, and then cells were treated with cycloheximide at the time of HSV-1 0^−^GFP inoculation. Thus, Ad-ICP0's capacity to stimulate ICP0^−GFP^ mRNA accumulation could be evaluated in the relative absence of other activators and/or repressors that formed in HSV-1 infected cells.

As expected, cells pre-treated with Ad-ICP0 expressed high levels of ICP0^−GFP^ mRNA despite the cycloheximide block to the synthesis of other HSV proteins (Fig. 3F, H). However, to our surprise, a similar result was observed in Ad-n212-treated cells treated with cycloheximide (Fig. 3G, H). Thus, when protein synthesis was blocked in HSV-infected cells, ICP0 was no longer required for the efficient synthesis of ICP0^−GFP^ mRNA (Fig. 3G). These findings argued against the hypothesis that ICP0 was required to actively *induce* ICP0^−GFP^ mRNA synthesis (Fig. 3A). Rather, the results suggested that ICP0 antagonized a repressor of the *ICP0^−GFP^* gene that was synthesized *de novo* in HSV-infected cells (Fig. 3B, green line). Further tests were conducted to explore the validity of this hypothesis.

### Testing the Predictions of a Repression-Antirepression Model of ICP0 Gene Regulation

Three predictions followed from an hypothesis that HSV-1 *ICP0* gene activity was regulated by a process of repression and antirepression: ***i.*** HSV-1 *ICP0* ^−^ and *ICP0^+^* viruses should express high and equivalent levels of ICP0 mRNA when synthesis of the repressor was blocked with cycloheximide (Fig. 3B, blue line); ***ii.*** an HSV-1 *ICP0^+^* virus should efficiently express ICP0 mRNA when the repressor accumulated in HSV-infected cells (Fig. 3B, green line); and ***iii.*** an HSV-1 *ICP0 ^−^* virus should exhibit little to no ICP0 mRNA synthesis when the repressor accumulated in the absence of ICP0 (Fig. 3B, red line).

**Figure 4:**
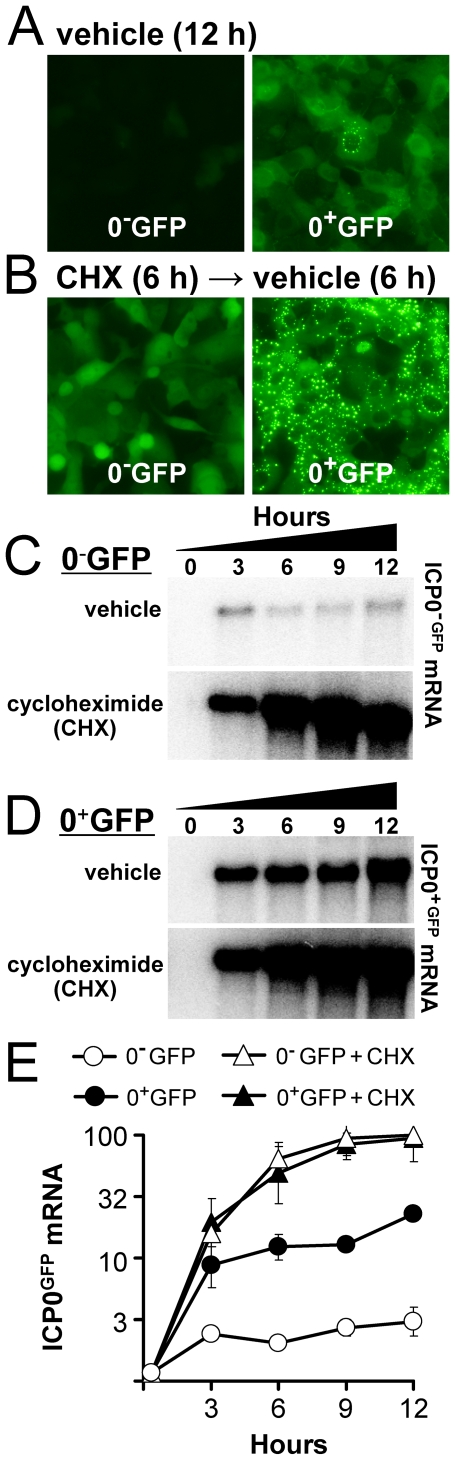
*De novo* repression of HSV *ICP0^GFP^* mRNA accumulation is antagonized by a GFP-tagged ICP0 protein. (A and B) Photomicrographs of ICP0^−GFP^ and ICP0^+GFP^ reporter protein fluorescence in Vero cells 12 hours after inoculation with 2.5 pfu per cell of HSV-1 0^−^GFP or 0^+^GFP (20× magnification). Cells were treated with either (A) medium containing no drug (vehicle) from 0 to 12 hours p.i., or (B) 200 µM cycloheximide from −0.5 to 6 hours p.i. followed by vehicle from 6 to 12 hours p.i. (C and D) Representative Northern blots of (C) ICP0^−GFP^ mRNA or (D) ICP0^+GFP^ mRNA harvested between 3 and 12 hours after inoculation with 2.5 pfu per cell of HSV-1 0^−^GFP or 0^+^GFP, respectively. Cells were treated with vehicle or 200 µM cycloheximide until the indicated time of RNA harvest (10 µg per lane). (E) Mean±sd of ICP0^−GFP^ and ICP0^+GFP^ mRNA levels plotted as a function of time of RNA harvest (n = 2 per time point per group).

As an initial test of these predictions, the relative expression of ICP0^−GFP^ or ICP0^+GFP^ fluorescent reporter proteins was compared in cells inoculated with 2.5 pfu per cell of HSV-1 0^−^GFP (*ICP0* ^−^) versus HSV-1 0^+^GFP (*ICP0* ^+^). In vehicle-treated cells, HSV-1 0^−^GFP expressed barely detectable levels of ICP0^−GFP^ fluorescence, whereas HSV-1 0^+^GFP expressed ICP0^+GFP^ fluorescence to readily detectable levels (Fig. 4A). In contrast, when protein synthesis was inhibited for the first 6 hours of infection with cycloheximide, then both ICP0^−GFP^ and ICP0^+GFP^ fluorescent proteins accumulated to high levels by 6 hours post-release from the cycloheximide block (Fig. 4B). Quantification by flow cytometry [40] demonstrated that ICP0^−GFP^ and ICP0^+GFP^ fluorescent proteins accumulated to high and equivalent levels following release from a cycloheximide block (Fig. S1).

The relative expression of ICP0^−GFP^ or ICP0^+GFP^ mRNA was compared in cells inoculated with HSV-1 0^−^GFP versus 0^+^GFP. When protein translation was allowed to occur, HSV-1 0^−^GFP produced levels of ICP0^−GFP^ mRNA that were barely detectable between 3 and 12 hours p.i. (Fig. 4C). When cycloheximide was used to block protein synthesis, ICP0^−GFP^ mRNA levels accumulated to levels that were ∼90 times background in HSV-1 0^−^GFP-infected cells (Fig. 4C, E). In contrast, the onset of protein translation did not prevent an *ICP0* ^+^ virus, HSV-1 0^+^GFP, from expressing ICP0^+GFP^ mRNA to levels that were ∼20 times background (Fig. 4D, E). When protein synthesis was inhibited with cycloheximide, HSV-1 0^+^GFP expressed ∼5-fold higher levels of ICP0^+GFP^ mRNA (Fig. 4D, E). As predicted, cycloheximide treatment allowed HSV-1 0^−^GFP and 0^+^GFP to express high and equivalent levels of ICP0^GFP^ mRNA (Fig. 4E). Control experiments verified that cycloheximide blocked HSV-1 L mRNA synthesis (i.e., glycoprotein D mRNA), but had only a negligible effect on cellular GAPDH mRNA levels (Fig. S2).

These findings were consistent with the predictions of a repression-antirepression model of *ICP0* gene regulation (Fig. 3B). However, questions remained about the identity of the *de novo* repressor that inhibited ICP0 mRNA synthesis in HSV-1 0^−^GFP-infected cells. Alternatively, it was possible that cycloheximide stimulated synthesis of all HSV mRNAs including ICP0^−GFP^ mRNA in a non-specific manner [41]. Further experiments were conducted to differentiate between these possibilities.

### Cycloheximide Stimulates HSV ICP0 Gene Expression in a VP16-Dependent Manner

**Figure 5:**
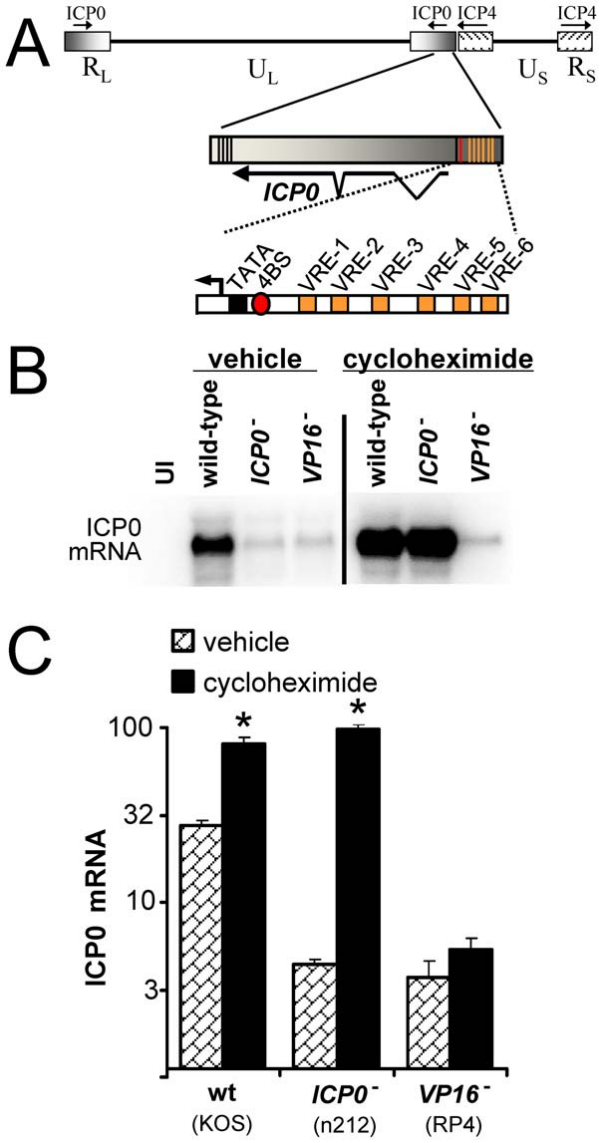
Cycloheximide increases *ICP0* gene expression in a VP16-dependent manner. (A) Schematic of *ICP0* promoter, which indicates the positions of six distal VP16-responsive elements (VREs), a proximal ICP4-binding site (4BS), and the TATA-box of the *ICP0* gene. (B) Representative Northern blot of ICP0 mRNA in Vero cells that were uninfected (UI) or were inoculated with 2.5 pfu per cell of HSV-1 KOS, n212 (*ICP0* ^−^), or RP4 (*VP16* ^−^), and which were treated with vehicle or 200 µM cycloheximide from −0.5 to 12 hours p.i., at which time total RNA was harvested (10 µg per lane). (C) Mean ± sem of ICP0 mRNA levels in KOS, n212, or RP4-infected cells (n = 3 per group). Asterisks denote significant differences between vehicle- and cycloheximide-treated cells inoculated with the same virus (p<0.01; two-tailed Student's t-test).

VP16 carried in HSV virions binds six VP16-responsive elements in the *ICP0* promoter at the outset of infection (Fig. 5A), and thus VP16 induces ICP0 mRNA synthesis [2], [42]. An experiment was conducted to determine if cycloheximide's effect on steady-state ICP0 mRNA levels was the result of a VP16-dependent process, or rather was due to a myriad of non-specific effects that should operate with equal efficiency in the presence or absence of VP16.

ICP0 mRNA accumulation was compared in cells inoculated with 2.5 pfu per cell of wild-type HSV-1 KOS, the *ICP0* ^−^ virus n212 [43], or the *VP16* ^−^ virus RP4 [44] (Table 1). When protein synthesis was allowed to occur, wild-type HSV-1 expressed ICP0 mRNA to levels that were 28-fold above background, whereas an *ICP0* ^−^ virus and *VP16* ^−^ virus expressed ICP0 mRNA to levels that were only 4-fold above background (Fig. 5B, C). When protein synthesis was blocked with cycloheximide, the *VP16* ^+^ viruses exhibited a robust increase in ICP0 mRNA levels; consequently, wild-type HSV and the *ICP0* ^−^ virus expressed high and equivalent levels of ICP0 mRNA in cycloheximide-treated cells (Fig. 5B, C). In contrast, the *VP16* ^−^ virus failed to efficiently express ICP0 mRNA in either vehicle- or cycloheximide-treated cells (Fig. 5C). Thus, cycloheximide was incapable of stimulating ICP0 mRNA accumulation in the absence of VP16. These data argued against the possibilities that cycloheximide might increase ICP0 mRNA abundance by ***1.*** decreasing the rate of ICP0 mRNA turnover, or by ***2.*** stimulating a non-specific increase in the rate of transcription of all genes, including the *ICP0* gene. Rather, the data were consistent with an hypothesis that cycloheximide blocked the formation of a *de novo* repressor of the *ICP0* gene that was dominant over the HSV-specific inducer of the *ICP0* gene, VP16.

### De Novo Repression of HSV ICP0 Gene Expression Is ICP4-Dependent

There is evidence that inhibition of protein synthesis causes all of HSV's IE mRNAs to be overexpressed [45]. One interpretation that has been offered is that cycloheximide's effect on HSV mRNA synthesis is global and non-specific [41]. However, there is a second, biologically important hypothesis that has not been adequately considered. As well as HSV ICP4's function as an essential activator of E and L mRNA transcription [46], ICP4 may function as a repressor of IE mRNA transcription [47]. To determine if ICP4 contributed to *de novo* repression of the *ICP0* gene, ICP0 mRNA accumulation was compared in cells inoculated with HSV-1 mutants deficient in the IE regulatory proteins ICP0, ICP27, ICP22, or ICP4 (Table 1).

**Figure 6:**
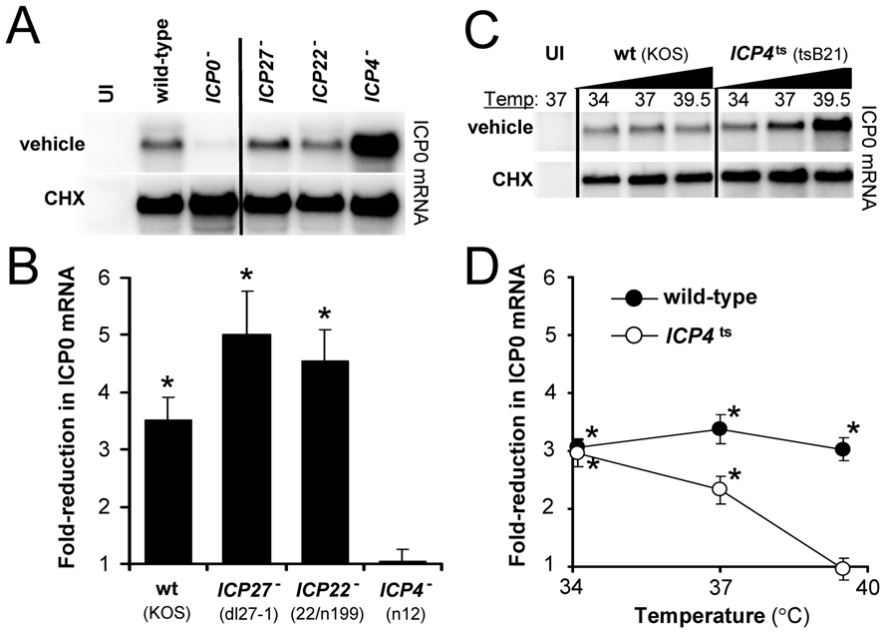
*De novo* repression of HSV *ICP0* gene expression is ICP4-dependent. (A) Representative Northern blot of ICP0 mRNA in Vero cells that were uninfected (UI) or were inoculated with 2.5 pfu per cell of HSV-1 KOS (wild-type), n212 (*ICP0* ^−^), dl27-1 (*ICP27* ^−^), 22/n199 (*ICP22* ^−^), or n12 (*ICP4* ^−^), which were treated with vehicle or 200 µM cycloheximide from −0.5 to 6 hours p.i., at which time total RNA was harvested (10 µg per lane). (B) Mean ± sem fold-reduction in ICP0 mRNA levels in vehicle-treated cells relative to cycloheximide-treated controls (fold-reduction = mRNA_CHX_÷mRNA_VEH_; n = 4 per group). (C) Representative Northern blot of ICP0 mRNA in Vero cells inoculated with 2.5 pfu per cell of KOS (*ICP4* ^+^) or HSV-1 tsB21 (*ICP4* ^ts^). Vehicle- and cycloheximide-treated cultures were incubated in CO_2_ chambers at 34.0, 37.0, or 39.5°C, and total RNA was harvested at 6 hours p.i. (10 µg per lane). (D) Mean ± sem fold-reduction in ICP0 mRNA levels plotted as a function of incubation temperature (fold-reduction = mRNA_CHX_÷mRNA_VEH_; n = 4 per group). In panels B and D, asterisks denote fold-reductions in ICP0 mRNA that significantly differ from the value of 1, which is predicted by a null hypothesis that cycloheximide will have no effect on ICP0 mRNA accumulation in HSV-infected cells (p<0.01; two-tailed Student's t-test).

Regardless of their different genotypes, 2.5 pfu per cell of each HSV-1 *VP16* ^+^ virus yielded high and equivalent levels of ICP0 mRNA in cycloheximide-treated cultures (Fig. 6A). The *ICP0* ^−^ virus, n212, expressed very low levels of ICP0 mRNA when protein translation was allowed to occur, whereas each of the *ICP0* ^+^ viruses expressed ICP0 mRNA to levels that were readily detectable in vehicle-treated cells (Fig. 6A). Wild-type HSV-1 KOS expressed 3.5±0.4-fold lower levels of ICP0 mRNA in vehicle-treated cells relative to cycloheximide-treated controls (Fig. 6A, B). Likewise, the *ICP27* ^−^ and *ICP22* ^−^ viruses, d27-1 [48] and 22/n199 [49], expressed 4- to 5-fold less ICP0 mRNA in vehicle-treated cells relative to cycloheximide-treated controls (Fig. 6A, B). These results suggested that the *de novo* repressor of ICP0 mRNA synthesis formed efficiently in the absence of ICP22, ICP27, or the ∼70 HSV E and L proteins whose synthesis was ICP27-dependent [48]. In contrast, an *ICP4* ^−^ virus, n12 [50], expressed high and equivalent levels of ICP0 mRNA in vehicle- and cycloheximide-treated cells (Fig. 6A, B). Therefore, *de novo* repression of ICP0 mRNA accumulation appeared to be dependent upon the synthesis of ICP4.

An independent test was conducted to corroborate this interpretation. Vero cells were inoculated with 2.5 pfu per cell of HSV-1 KOS (*ICP4* ^+^) or HSV-1 tsB21 (*ICP4* ^ts^), which bears a temperature-sensitive lesion in the C-terminus of ICP4; this mutation renders ICP4 non-functional at a temperature of 39.5°C [47]. At 34.0°, 37.0°, and 39.5°C, wild-type HSV-1 KOS expressed ∼3-fold less ICP0 mRNA in vehicle-treated cells relative to cycloheximide-treated controls (Fig. 6C, D). Likewise, HSV-1 tsB21 expressed 2.9- and 2.3-fold less ICP0 mRNA in vehicle-treated cells at 34.0° and 37.0°C, respectively, relative to cycloheximide-treated controls (Fig. 6C, D). In contrast, at the non-permissive temperature of 39.5°C, HSV-1 tsB21 expressed equivalent levels of ICP0 mRNA in both vehicle- and cycloheximide-treated cells (Fig. 6C, D). Collectively these results indicated that *de novo* repression of ICP0 mRNA accumulation was dependent upon the synthesis of a biologically active form of HSV's major transcriptional regulator, ICP4.

### De Novo Repression of HSV ICP0^GFP^ Reporter Genes Is ICP4-Binding-Site-Dependent

**Figure 7:**
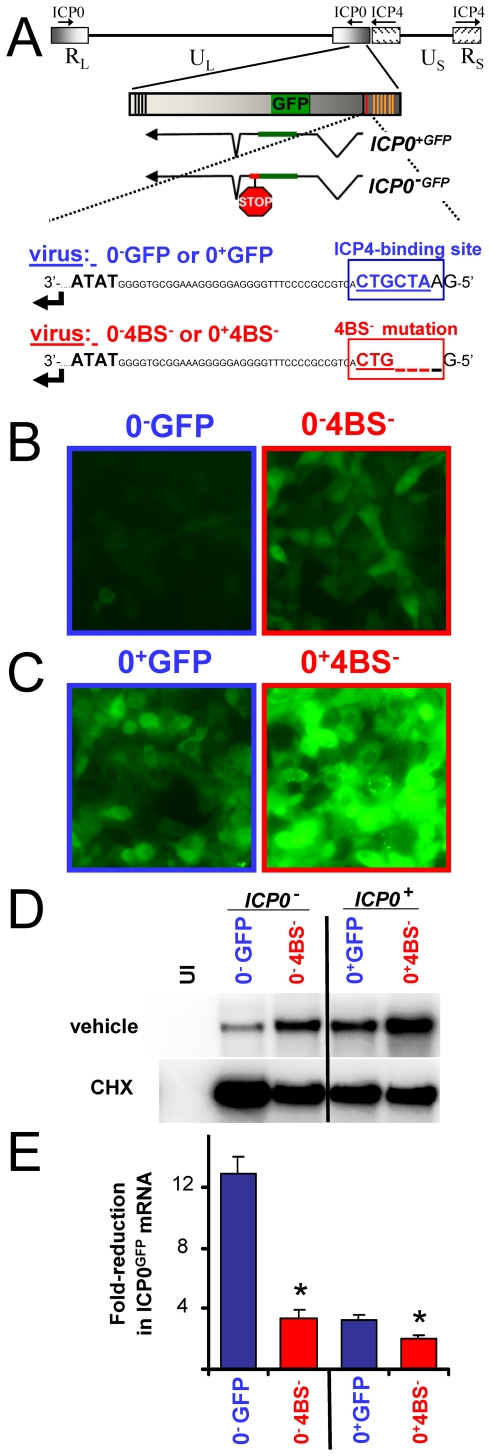
*De novo* repression of *ICP0^GFP^* gene expression is ICP4-binding-site-dependent. (A) Schematic of the ICP4 DNA-binding site (4BS) in HSV-1 0^−^GFP and 0^+^GFP versus the mutated 4BS-element in HSV-1 0^−^4BS^−^ and 0^+^4BS^−^. All four viruses transcribe 3.5 kb ICP0^GFP^ mRNAs, but HSV-1 0^−^4BS^−^ and 0^+^4BS^−^ are deleted of bases −43 to −40 relative to the TATA box of the *ICP0^GFP^* gene, which disrupts the consensus ICP4-binding site, 5′-ATCGTC-3′. (B and C) Photomicrographs of ICP0^−GFP^ and ICP0^+GFP^ fluorescent protein accumulation in Vero cells 12 hours after inoculation with 5 pfu per cell of (B) HSV-1 0^−^GFP versus 0^−^4BS^−^ or (C) HSV-1 0^+^GFP versus 0^+^4BS^−^ (20× magnification). (D) Representative Northern blots of ICP0^GFP^ mRNA accumulation in Vero cells that were uninfected (UI) or were inoculated with 5 pfu per cell of HSV-1 0^−^GFP (*ICP0* ^−^), 0^−^4BS^−^ (*ICP0* ^−^), 0^+^GFP (*ICP0* ^+^), or 0^+^4BS^−^ (*ICP0* ^+^), and which were treated with vehicle or 200 µM cycloheximide from −0.5 to 12 hours p.i., at which time total RNA was harvested (10 µg per lane). (E) Mean ± sem fold-reduction in ICP0^GFP^ mRNA levels in vehicle-treated cells relative to cycloheximide-treated controls (fold-reduction = mRNA _CHX_÷mRNA _VEH_; n = 3 per group). Asterisks denote significant differences in fold-reduction in ICP0^GFP^ mRNA levels between the *ICP0* ^−^ viruses, HSV-1 0^−^GFP versus 0^−^4BS^−^ (p<0.001), and between the *ICP0* ^+^ viruses, HSV-1 0^+^GFP versus 0^+^4BS^−^ (p<0.01; two-tailed Student's t-test).

A consensus ICP4-binding site (ATCGTC) occurs 40-bp upstream of the TATA box in the *ICP0* promoter [37], [51]. To determine if ICP4 binding to the *ICP0* promoter contributed to *de novo* repression of ICP0^GFP^ mRNA synthesis, the recombinant viruses HSV-1 0^−^4BS^−^ and 0^+^4BS^−^ were constructed which were equivalent to HSV-1 0^−^GFP and 0^+^GFP, respectively, but which bore a 4-bp deletion in the ICP4-binding site in the *ICP0* promoter (Fig. 7A).

A test was conducted to determine if disruption of the ICP4-binding site might alleviate *de novo* repression of the *ICP0^−GFP^* gene. Vero cells were inoculated with 5 pfu per cell of HSV-1 0^−^GFP or HSV-1 0^−^4BS^−^, and ICP0^−GFP^ fluorescence was compared at 12 hours p.i. (Fig. 7B). In cells inoculated with HSV-1 0^−^GFP, ICP0^−GFP^ fluorescence was barely visible (Fig. 7B). In contrast, ICP0^−GFP^ fluorescence was readily detected in cells inoculated with HSV-1 0^−^4BS^−^ (Fig. 7B). Likewise, ICP0^+GFP^ fluorescence accumulated to appreciably higher levels in cells inoculated with HSV-1 0^+^4BS^−^ relative to HSV-1 0^+^GFP (Fig. 7C). These observations suggested that disruption of the proximal ICP4-binding site in the *ICP0* promoter significantly alleviated *de novo* repression of the *ICP0^−GFP^* and *ICP0^+GFP^* genes.

To corroborate this interpretation, accumulation of the 3.5 kb ICP0^GFP^ RNA species was compared in cells inoculated with 5 pfu per cell of HSV-1 0^−^GFP, 0^−^4BS^−^, 0^+^GFP, or 0^+^4BS^−^ (Fig. 7D). Each virus yielded high levels of ICP0^−GFP^ or ICP0^+GFP^ mRNA in cycloheximide-treated cultures (Fig. 7D). HSV-1 0^−^GFP exhibited a 13±1-fold repression of ICP0^−GFP^ mRNA synthesis in vehicle-treated cells relative to cycloheximide-treated controls (Fig. 7D, 7E). Disruption of the ICP4-binding site in the *ICP0* promoter alleviated *de novo* repression by 4-fold, and thus HSV-1 0^−^4BS^−^ exhibited only a 3.3±0.5-fold reduction in ICP0^−GFP^ mRNA in vehicle-treated cells (Fig. 7D, 7E). Disruption of the ICP4-binding site in the *ICP0^−GFP^* gene increased the efficiency of ICP0^GFP^ mRNA synthesis to the same extent as synthesis of a biologically active ICP0 protein; thus, HSV-1 0^−^4BS^−^ and 0^+^GFP both exhibited a 3.3-fold reduction in ICP0^GFP^ mRNA levels in vehicle-treated cells relative to their respective cycloheximide-treated controls (Fig. 7D, 7E). Disruption of the ICP4-binding site in the *ICP0* promoter of HSV-1 0^+^4BS^−^ resulted in a significant de-repression; thus ICP0^+GFP^ mRNA levels in vehicle-treated cells infected with HSV-1 0^+^4BS^−^ were only 2.1±0.2-fold lower than cycloheximide-treated controls (Fig. 7D, 7E). Collectively, these observations indicated that the magnitude of *de novo* repression of ICP0^GFP^ mRNA synthesis was significantly dependent upon the ICP4-binding site proximal to the transcriptional start site of the *ICP0* gene (Fig. 7A).

### Adenovirus-Encoded ICP4 Substitutes for the De Novo Repressor of the HSV ICP0^−GFP^ Gene

Our results raised the possibility that the *de novo* repressor of ICP0 mRNA synthesis was not a cellular protein, but rather was the HSV ICP4 protein because ***1.*** loss of ICP4 function de-repressed ICP0 mRNA synthesis to the same extent as cycloheximide treatment (Fig. 6), and ***2.*** a 4-bp deletion that removed a single ICP4-binding site significantly alleviated *de novo* repression of ICP0 mRNA synthesis (Fig. 7). Based on this hypothesis, it was predicted that pre-treatment of Vero cells with an ICP4-expressing adenovirus should negate cycloheximide's capacity to stimulate ICP0^−GFP^ mRNA synthesis in HSV-1 0^−^GFP-infected cells.

**Figure 8:**
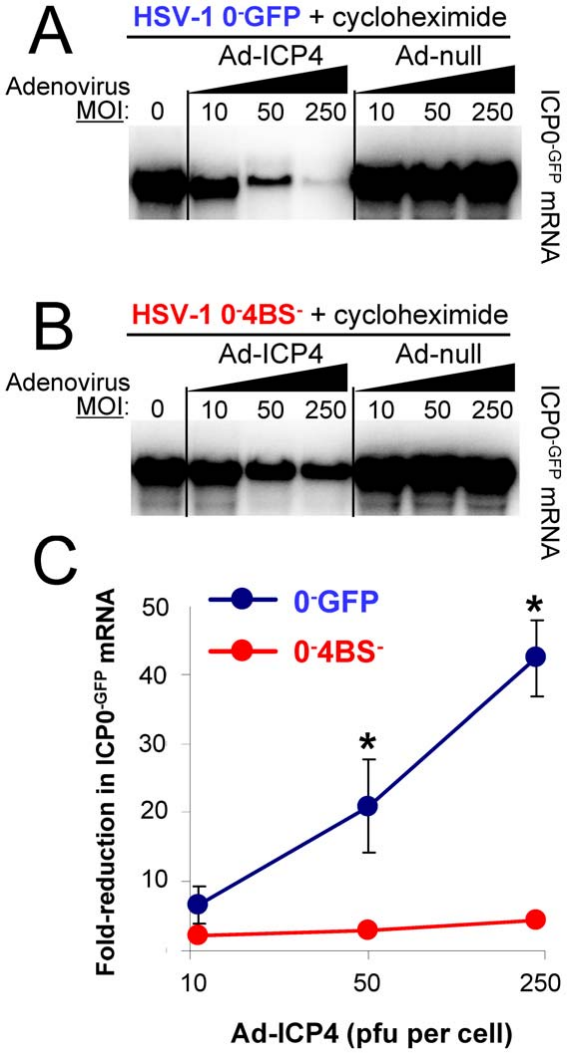
Exogenous ICP4 substitutes for the *de novo* repressor of the *ICP0^−GFP^* gene. Representative Northern blots of ICP0^−GFP^ mRNA levels in Vero cells treated with 200 µM cycloheximide and inoculated with 5 pfu per cell of (**A**) HSV-1 0^−^GFP or (**B**) HSV-1 0^−^4BS^−^ (10 µg total RNA per lane; time of harvest = 12 hours p.i.). Twelve hours prior to HSV-1 inoculation, cells were pre-treated with 0, 10, 50, or 250 pfu per cell of Ad-ICP4 or Ad-null whose TRE promoters were induced with 20 pfu per cell of Ad.CMV-TetOn and 10 µM doxycycline. (**C**) Mean ± sem fold-reduction in ICP0^−GFP^ mRNA levels in HSV-1 0^−^GFP or 0^−^4BS^−^ infected cells as a function of the MOI of Ad-ICP4 in the pre-treatment (n = 3 per group). Asterisks denote significant differences in Ad-ICP4-induced reductions in ICP0^−GFP^ mRNA levels between HSV-1 0^−^GFP and 0^−^4BS^−^ (p<0.001; two-way analysis of variance).

To test this prediction, Vero cells were pre-loaded with 0 to 250 pfu per cell of Ad-ICP4 or a null control vector, and cells were inoculated 12 hours later with HSV-1 0^−^GFP in the presence of cycloheximide. As expected, cells that received no adenovirus vector and which were inoculated with HSV-1 0^−^GFP in the presence of cycloheximide expressed high levels of ICP0^−GFP^ mRNA (Fig. 8A). However, when Vero cells were pre-treated with Ad-ICP4, cycloheximide's capacity to stimulate ICP0^−GFP^ mRNA synthesis dissipated and this inhibition was adenovirus dose-dependent (Fig. 8A). Despite cycloheximide treatment, the highest MOI of Ad-ICP4 caused a 42-fold reduction in ICP0^−GFP^ mRNA levels in HSV-1 0^−^GFP-infected cells (blue line in Fig. 8C). This was not a non-specific effect of the adenovirus vector, as MOIs of 10 to 250 pfu per cell of Ad-null did not reduce the efficiency of ICP0^−GFP^ mRNA synthesis in HSV-1 0^−^GFP-infected cells treated with cycloheximide (Fig. 8A).

To verify that Ad-ICP4's capacity to repress ICP0^−GFP^ mRNA synthesis was ICP4-binding-site-dependent, the experiment was performed in parallel with HSV-1 0^−^4BS^−^. Ad-ICP4 had a modest effect on ICP0^−GFP^ mRNA synthesis in HSV-1 0^−^4BS^−^-infected cells (Fig. 8B). However, the potency of inhibition was 10-fold less than observed in HSV-1 0^−^GFP-infected cells (red line in Fig. 8C). Specifically, an MOI of 250 pfu per cell of Ad-ICP4 produced only a 4.4-fold reduction in ICP0^−GFP^ mRNA levels in HSV-1 0^−^4BS^−^-infected cells treated with cycloheximide (Fig. 8C). Therefore, adenovirus-encoded ICP4 was sufficient to substitute for the *de novo* repressor of the *ICP0^−GFP^* gene that routinely formed in HSV-1 0^−^GFP infected cells.

### ICP0 Physically Interacts with the Repressor of Its Gene, ICP4, in HSV-Infected Cells

A physical interaction between ICP0 and ICP4 might begin to explain how ICP0 influences ICP4's activity as a transcriptional regulator. The available evidence from far-Western blotting [52] and co-localization studies [53] suggested that ICP0 may physically interact with ICP4. However, direct evidence was lacking to address whether or not ICP0 and ICP4 physically interact in the context of HSV-infected cells.

**Figure 9:**
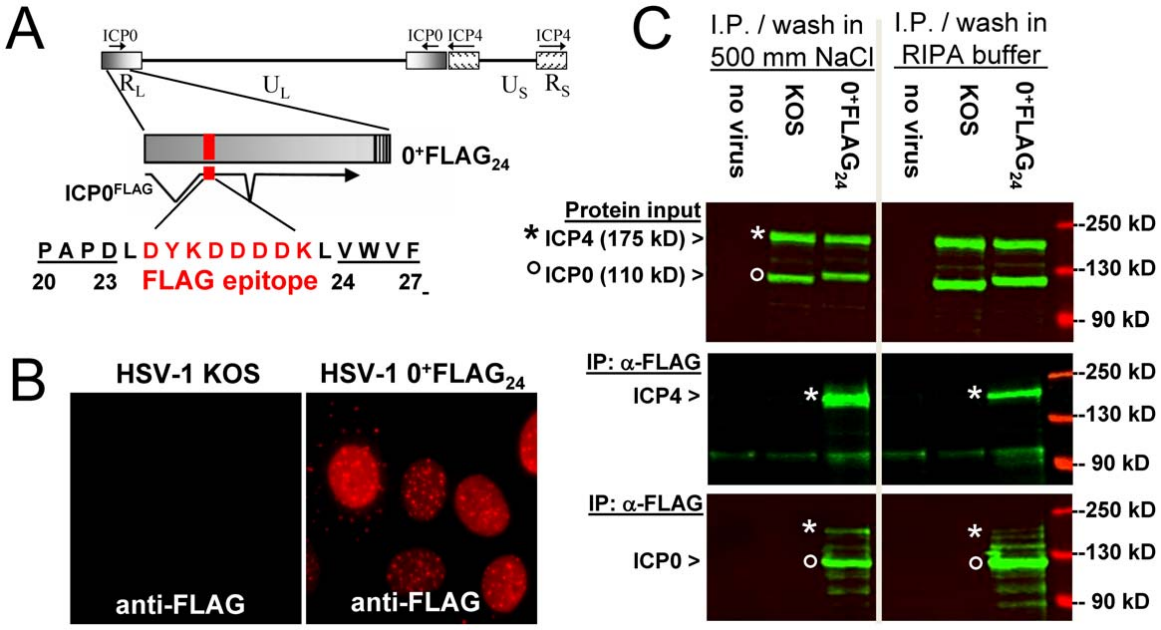
ICP4 co-immunoprecipitates with FLAG-tagged ICP0. (A) Schematic of 30-bp insertion in the HSV-1 recombinant virus, 0^+^FLAG_24_, which places a FLAG tag coding sequence between codons 23 and 24 of the *ICP0* gene. (B and C) Vero cells were inoculated with 5 pfu per cell of HSV-1 KOS (wild-type) or HSV-1 0^+^FLAG_24_, which were treated with 200 µM cycloheximide from −0.5 to 10 hours p.i., released into actinomycin D (10 µg/ml) from 10 to 16 hours p.i., and harvested for immunofluorescent staining or immunoprecipitation. (B) Immunofluorescent staining with a FLAG-specific antibody and AlexaFluor 594-conjugated secondary antibody. (C) Cells were harvested in an NP40-based buffer containing 150 mM NaCl (left panel) or RIPA buffer (right panel). The relative abundance of ICP4 and ICP0 was compared in 5% of total cell lysates (top panel), and the remainder was immunoprecipitated with anti-FLAG agarose. Immunoprecipiates were washed in 500 mM NaCl (left panel) or RIPA buffer (right panel) prior to electrophoresis and blotting, and were probed with ICP4-specific monoclonal antibody 58S (middle panel), followed by re-probing with ICP0-specific monoclonal antibody H1083 (bottom panel). The relative locations of ICP4 and ICP0 are denoted by asterisks and open circles, respectively.

To address this gap in knowledge, a recombinant virus was constructed that bore a FLAG epitope between amino acids 23 and 24 of ICP0, and which is referred to as HSV-1 0^+^FLAG_24_ (Fig. 9A). Southern blot analysis validated that the FLAG coding sequence was inserted in both copies of the *ICP0* gene, and that HSV-1 0^+^FLAG_24_ grew with wild-type efficiency (not shown). Immunofluorescent staining verified that HSV-1 0^+^FLAG_24_ expressed a FLAG-tagged protein that exhibited a nuclear-staining pattern typical of ICP0 during the IE phase of HSV infection (Fig. 9B; 10 hours in cycloheximide→6 hours in actinomycin D).

Immunoprecipitation experiments were performed to determine if ICP4 physically interacted with FLAG-tagged ICP0. Vero cells were inoculated with vehicle or 5 pfu per cell of HSV-1 KOS or 0^+^FLAG_24_ in the presence of cycloheximide (0–10 hours p.i.) followed by actinomycin D (10–16 hours p.i.), such that only viral IE proteins were efficiently synthesized. Input levels of ICP4 and ICP0 in each immunoprecipitation reaction were equivalent, as verified by Western blot analysis with monoclonal antibodies 58S and H1083, respectively (Fig. 9C, top panel). The remainder of each protein sample was immunoprecipitated with an anti-FLAG antibody-agarose conjugate under conditions of medium or high stringency, and immunoprecipitates were analyzed for the presence of ICP4.

Significant amounts of ICP4 co-immunoprecipitated with FLAG-tagged ICP0 from lysates of HSV-1 0^+^FLAG_24_-infected cells (Fig. 9C, middle panel). Non-specific pull down of ICP4 was negligible from lysates of uninfected Vero cells or KOS-infected cells (Fig. 9C). Therefore, the α-FLAG agarose only appeared to pull down ICP4 when the FLAG epitope was present in ICP0. Consistent with this interpretation, re-probing of the same blot with an ICP0-specific monoclonal antibody, H1083, verified that anti-FLAG agarose efficiently pulled down the 110 kDa FLAG-tagged ICP0, but did not pull down wild-type ICP0 (Fig. 9C, lower panel). We conclude that ICP0 and ICP4 do indeed physically interact in HSV-infected cells, and propose that this interaction is likely relevant in explaining how ICP0 antagonizes ICP4's capacity to silence ICP0 mRNA synthesis from the HSV genome.

## Discussion

### Identification of an ICP0-Antagonized Repressor of HSV mRNA Synthesis

HSV routinely establishes latent infections in its human host. During the latent phase of infection, most of the HSV genome is transcriptionally silent. It is unclear how the HSV genome is silenced, but it is generally agreed that synthesis of ICP0 destabilizes repression and thus triggers reactivation of mRNA synthesis from latent HSV genomes [7], [8], [54].

The search for an ICP0-antagonized transcriptional repressor has led investigators to consider the possibility that PML and Sp100 [17], IRF-3 and IRF-7 [25], and centromere proteins CENP-B and CENP-C [26], [27] may represent the repressors of the HSV genome that are antagonized by ICP0. Although ICP0 may alter the subcellular distribution, stability, or function of these proteins, there is no direct evidence that these proteins are capable of restricting mRNA synthesis from the HSV genome. Likewise, it has been postulated that epigenetic silencing may be the ICP0-antagonized repressive mechanism that is capable of silencing the HSV genome [20], [55]. However, no specific histone deacetylase or histone mark has been associated with more than a 2-fold reduction in HSV mRNA synthesis [18], [19], [56], [57].

Although an ICP0-antagonized repressor has not been identified, it would be reasonable to expect that the repressive entity: ***1.*** should be necessary and sufficient to restrict the synthesis of one or more HSV mRNAs; ***2.*** should be unable to silence HSV mRNA synthesis when ICP0 accumulates; and ***3.*** may physically interact with ICP0. The results of the current study establish that HSV's major transcriptional regulator, ICP4, meets these criteria, and thus appears to be the first *bona fide* ICP0-antagonized repressor of HSV mRNA synthesis.

The results clarify that ICP4 is capable of silencing mRNA synthesis from the *ICP0* gene when ICP0 fails to accumulate. At low MOIs, HSV *ICP0* ^−^ viruses establish quiescent infections in 99% of infected cells [9], [10]. Intriguingly, ICP4 accumulates to detectable levels in >85% of cells that become quiescently infected with HSV *ICP0* ^−^ viruses [58]. Thus, further studies will be required to determine if ICP4 routinely accumulates in the absence of ICP0 in HSV quiescently infected cells, and if so, to determine if ICP4 plays a causal role in restricting mRNA synthesis from quiescent HSV genomes [59], [60], [61].

### Differential Regulation of ICP0 mRNA Synthesis in HSV-Infected Cells

There is anecdotal evidence that the *ICP0* gene is differentially regulated in HSV-infected cells, but it remains unclear how such regulation is achieved. The HSV latency-associated transcripts (LATs) have been discussed for their potential to repress *ICP0* gene expression [62], [63]. Likewise, VP16's capacity to function as a transactivator of IE genes has been considered as a potential target of differential regulation [64], [65], [66]. However, it remains unclear that LATs or VP16 are sufficient to explain how the *ICP0* gene is regulated.

The ICP4-binding site in the *ICP0* promoter was identified 20 years ago as a potential means by which ICP4 might regulate ICP0 mRNA synthesis [51]. When studied in the context of HSV *ICP0* ^+^ viruses, some investigators concluded that ICP4's effect on *ICP0* gene expression was negligible [36]. Other investigators noted that ICP4 produced a modest decrease in ICP0 mRNA levels in the context of *ICP0* ^+^ viruses [37], but ICP4's full potential to silence *ICP0* gene expression went unrecognized.

The current study demonstrates that the *ICP0* gene is differentially regulated by three HSV proteins, VP16, ICP4, and ICP0, whose precise relationship to one another does not appear to be fully resolved. Functionally, the results suggest that VP16 and ICP4 ‘push’ ICP0 mRNA synthesis in opposite directions, and accumulation of ICP0 dictates which way this equilibrium will tilt. When ICP0 fails to accumulate, ICP4 is capable of silencing ICP0 mRNA synthesis despite VP16 in the tegument of virions (Fig. 3–6; *ICP0* ^−^ treatments). When formation of the *de novo* repressor is blocked with cycloheximide, VP16's full capacity to transactivate the HSV-1 *ICP0* gene is revealed (Fig. 5). If ICP0 accumulates, ICP4 fails to efficiently repress ICP0 mRNA synthesis (Fig. 3–6; *ICP0* ^+^ treatments). These findings suggest that a hierarchy exists in the HSV regulatory scheme in which ***i.*** ICP0-dependent antirepression of the *ICP0* gene is dominant over ***ii.*** ICP4-dependent repression of the *ICP0* gene, which is dominant over ***iii.*** VP16-dependent induction of the *ICP0* gene. Therefore, while VP16 induces transcription of both the *ICP4* and *ICP0* genes, the opposing processes of ICP4-dependent repression and ICP0-dependent antirepression appear to dictate whether the *ICP0* gene remains ON or OFF in HSV-infected cells (Fig. 3B).

### ICP4-Dependent Silencing of the ICP0 Gene: A Cell Type-Specific Phenomenon?

Less than 1 HSV *ICP0* ^−^ virus per 10,000 pfu is able to form plaques in monolayers of HEL cells [67]. In contrast, 1 HSV *ICP0* ^−^ virus per 100 pfu forms plaques in Vero cell monolayers [9], and nearly 100% of HSV *ICP0* ^−^ viruses form plaques in U2OS cell monolayers [52]. The highly variable permissivity of host cells for HSV *ICP0* ^−^ viruses raises the question, *“Is ICP4's capacity to silence ICP0 gene expression in Vero cells representative of what occurs in other cell types?”* Therefore, it is noteworthy that HSV *ICP0* ^−^ viruses form plaques with an efficiency that is indistinguishable from Vero cells in 11 of 15 other cell lines tested to date. Specifically, 0.5–2% of HSV *ICP0* ^−^ viruses form plaques in monolayers of human 293 cells, mouse 3T3 cells, hamster BHK-21 cells, bovine BIEC cells, bovine BUVEC cells, human Caco-2 cells, human HeLa cells, monkey MA104 cells, canine MDCK cells, pig PK-15 cells, and human WiDr cells (unpublished data of W. Halford). Further testing will be required to determine if the phenomenon of ICP4-dependent silencing of the *ICP0* gene varies in proportion to the degree with which HSV *ICP0* ^−^ viral replication is restricted in different cell lines.

### ICP0 as a Regulatory Subunit of ICP4: A Missing Lynchpin in HSV Gene Regulation?

The 1298-amino-acid ICP4 protein functions as an essential activator that recruits cellular TATA-binding protein complexes to HSV genomes, and thus stimulates mRNA synthesis from HSV E and L genes [46]. ICP4 also possesses a second activity as a repressor of HSV IE genes, which requires the binding of 350 kDa ICP4 homodimers to consensus ATCGTC sequences [68], [69]. To date, it remains unclear how the balance between ICP4's opposing transcriptional repressor versus activator functions is controlled.

Since the discovery that ICP0 re-organizes PML nuclear bodies [29], ICP4 and ICP0 have been predominantly studied in isolation from one another. However, ICP0 was initially discovered as a factor that stimulated a 20-fold increase in ICP4-dependent mRNA synthesis from HSV E and L promoters [6]. The results of the current study establish that ICP0 and ICP4 participate in a robust physical interaction in HSV-infected cells (Fig. 9). We postulate that ICP0's physical interaction with ICP4 is likely regulatory in nature, and hence may be relevant in explaining how ICP0 potentiates ICP4-dependent E and L mRNA synthesis [5], [6], as well as how ICP0 antagonizes ICP4-dependent repression of ICP0 mRNA synthesis (Fig. 3–5). Therefore, we propose that the accumulation of ICP0 in HSV-infected cells (or lack thereof) may dictate whether ICP4 functions predominantly as an activator, or a repressor, of HSV mRNA synthesis. Further studies will be required to test the validity of this important hypothesis.

### How Does ICP0 Antagonize ICP4-Dependent Repression?

What specific protein-DNA complexes explain how ICP0 antagonizes ICP4-dependent silencing of the *ICP0* gene? The mechanisms that underlie the phenomenon documented herein are unknown, and further study will be required to differentiate between many possibilities. To illustrate this point, we present two molecular models that are each consistent with our findings, but which suggest two completely different interpretations for the significance of an interaction between ICP0 and ICP4 in the biology of HSV infections.

The first model, the ICP0-ICP4 Interference Model, is the most intuitively obvious interpretation and may reflect the mental model that many readers have invoked up to this point in the manuscript to make sense of the results. Under an ICP0-ICP4 Interference Model, it could be predicted that ***1.*** ICP4 binding to HSV dsDNA serves the sole purpose of repressing mRNA synthesis from adjacent HSV genes, and that ***2.*** ICP0 blocks ICP4's capacity to bind dsDNA, and thus ICP0 blocks ICP4-dependent repression of the HSV *ICP0* gene. However, synthesis of ICP4 is required for synthesis of viral E and L mRNAs, and mutations that disrupt ICP4's DNA-binding domain also render HSV incapable of synthesizing its ∼70 E and L mRNAs that are required for HSV replication [50], [70], [71]. Therefore, it is difficult to envision how an ICP0-based activity that dislodges ICP4 from dsDNA would help explain how ICP0 potentiates ICP4-dependent synthesis of HSV E and L mRNAs [5], [6].

An ICP0-ICP4 Synergy Model is less intuitively obvious, but is more consistent with ICP0's capacity to enhance ICP4-dependent synthesis of HSV E and L mRNAs. Under this latter model, it may be envisioned that ***1.*** the functional purpose of ICP4 and the >50 ICP4-binding sites that span the HSV genome is to allow ICP4 to form a histone-like scaffold that coats HSV genomes; ***2.*** the 1298 amino-acid ICP4 protein possesses numerous domains that allow ICP4 to interact with cellular proteins [72], [73] and viral proteins (Fig. 9) which influence whether ICP4 stimulates or represses mRNA synthesis from the underlying HSV DNA; and ***3.*** the nature of ICP0's interaction with ICP4 relates to potentiating ICP4-dependent activation of mRNA synthesis from HSV DNA. Clearly, further investigation will be required to determine if the specific protein-DNA interactions that occur in HSV-infected cells are consistent with an ICP0-ICP4 Synergy Model of HSV gene regulation.

### Conclusion

The herpesviruses and temperate bacteriophage are large dsDNA viruses that share the capacity to alternate between productive and silent infections. Many similarities have emerged in the mechanisms that herpesviruses and the tailed bacteriophage employ to replicate and encapsidate their large dsDNA genomes [74], [75], [76], [77]. Such observations point to a common evolutionary ancestry between these two ancient families of viruses [78], [79]. We conclude that the results of the current study appear to add to this growing list of inexplicable similarities.

The parallel capacity of HSV and bacteriophage λ to alternate between productive and silent infections may not be a coincidence. Rather, both viruses may control the onset of viral replication through a system of repression and antirepression of a few IE proteins whose accumulation, or lack thereof, dictates the probability of replication. In λ phage, the ‘decision’ between productive versus silent infections hinges upon whether the λ *cro antirepressor* gene is expressed or repressed [80], [81], [82]. Thus, important questions follow from the observation that HSV encodes its own transcriptional repressor function, embedded in ICP4, which is capable of silencing the HSV *antirepressor* gene that encodes ICP0. Further study will be required to determine if, in fact, repression-antirepression of the *ICP0* gene is relevant to the natural process by which HSV ‘decides’ whether a given infection will be productive or silent.

## Materials and Methods

### Cells and Viruses

Vero cells and 293 cells were obtained from the American Type Culture Collection (Manassas, VA). ICP4-complementing E5 and ICP0-complementing L7 cell lines were kindly provided by Neal Deluca (University of Pittsburgh; Ref. [83]). ICP27-complementing V27 cells and HSV-1 d27-1 (*ICP27* ^−^) virus were a kind gift of Steve Rice (University of Minnesota Medical School, Minneapolis; Ref. 48). Cell lines were propagated in Dulbecco's Modified Eagle's medium supplemented with 5% fetal bovine serum and antibiotics.

Table 1 summarizes the HSV-1 recombinant viruses used in this study, which are derivative of low passage strains of wild-type HSV-1 strain KOS [84]. HSV-1 KOS, HSV-1 22/n199 (*ICP22* ^−^; Ref. 49), HSV-1 0^+^GFP (*ICP0^+GFP^*), and HSV-1 0^+^4BS^−^ (*ICP0^+GFP^*) were propagated in Vero cells. The HSV-1 *ICP4* ^−^ virus n12 [50] and the ICP4 temperature-sensitive virus HSV-1 tsB21 [47] were propagated in ICP4-complementing E5 cells. The HSV-1 *ICP0* ^−^ viruses n212 [43], 0^−^GFP (*ICP0^−GFP^*), and 0^−^4BS^−^ (*ICP0^−GFP^*) were propagated and titered in ICP0-complementing L7 cells, as was the HSV-1 *VP16* ^−^ virus RP4 [44] that was kindly provided by Steve Triezenberg (Michigan State University, East Lansing).

The salient features of each mutation in the *ICP0* gene are summarized, as follows: ***i.*** the HSV-1 *ICP0* ^−^ null virus n212 contains a 14 bp translational stop insertion (ctagactagtctag) in codon 212 of the *ICP0* gene; hence, the n212 virus encodes the N-terminal 211 of 775 amino acids of ICP0 [43]; ***ii.*** the HSV-1 *ICP0* ^−^ null viruses 0^−^GFP and 0^−^4BS^−^ each contain a *GFP* open-reading frame and translational stop inserted in codon 105 of the *ICP0* gene; hence, HSV-1 0^−^GFP and 0^−^4BS^−^ encode the N-terminal 104 amino acids of ICP0 fused to GFP; and ***iii.*** the HSV-1 *ICP0* ^+^ viruses 0^+^GFP and 0^+^4BS^−^ each encode a chimeric protein in which GFP is inserted between amino acids 104 and 105 of ICP0.

Table 2 summarizes the ΔE1–ΔE3 adenovirus vectors used in this study, which were propagated in human embryonic kidney 293 cells in complete DMEM, as described previously [7]. Adenovirus titers were determined by a limiting dilution analysis in 96-well cultures of 293 cells to establish the titer of infectious virus in terms of 50% tissue-culture infectious dose (TCID_50_). One TCID_50_ unit is equivalent to 1 pfu, but the TCID_50_ assay is less prone to underestimate adenovirus vector titers (unpublished data of W. Halford).

### Plasmid Precursors of HSV-1 Recombinant Viruses

The construction of plasmid precursors of the HSV-1 recombinant viruses used in this study is described. A 7.3 kb DNA fragment which encompasses the entire *LAT-ICP0* locus (118,003–125,269; accession number X14112) was subcloned from HSV-1 (strain KOS) into a pCRII plasmid vector (Invitrogen Corporation, Carlsbad, CA). All mutations in the *ICP0* gene were subcloned into this plasmid, pCRII-HSV118-125, and plasmids bearing the desired mutation were co-transfected with HSV-1 strain KOS to obtain each of the recombinant viruses described below. The mutagenesis steps taken to create each mutant allele of the *ICP0* gene are summarized, as follows.

- ***p0^−^ GFP*** **:** The plasmid p0^−^GFP was created by a multi-step sub-cloning procedure, which inserted a polylinker, *GFP* coding sequence, and stop codon derived from the plasmid peGFP-N1 (Clontech Laboratories) into a Xho I site in codon 104 of the *ICP0 gene*. The resulting plasmid, p0^−^GFP, encoded the N-terminal 104 amino acids of ICP0 fused to GFP by a 14-amino-acid linker. Homologous recombination between HSV-1 KOS and the plasmid p0^−^GFP yielded the recombinant virus HSV-1 0^−^GFP (Table 1).
- ***p0^+^ GFP*** **:** The plasmid p0^+^GFP was derived by subcloning a dsDNA linker containing BsrGI and Xho I sites ( tgtaca agatat ctcgag ) into the 3′ end of the *GFP* coding sequence in p0^−^GFP. This sub-cloning step deleted 7 bp, thus removing a TAA terminator codon and placing the *GFP* and *ICP0* coding sequences in the same open-reading frame. The resulting plasmid, p0^+^GFP, encoded the ICP0^+*GFP*^ reporter protein, which bears a GFP insertion between amino acids 104 and 105 of ICP0. Homologous recombination between HSV-1 KOS and p0^+^GFP yielded the recombinant virus HSV-1 0^+^GFP (Table 1).
- ***p0^−^ 4BS^−^ and p0^+^ 4BS^−^*** **:** The plasmids p0^−^4BS^−^ and p0^+^4BS^−^ were created as follows. A plasmid, pmin0^−^GFP, contains the *ICP0* promoter region and a unique Tfi I restriction site that overlaps the ICP4 DNA-binding site (4BS) element in the *ICP0* promoter. Thus, mutation of the 4BS element was achieved by linearizing pmin0^−^GFP with Tfi I, using mung bean exonuclease to remove ssDNA overhangs, and religating with T4 DNA ligase. The 4-bp deletion in the 4BS element was confirmed by the loss of the Tfi I restriction site and DNA sequencing (Fig. 7A). The ICP4-binding site mutation was transferred into the larger plasmids p0^−^GFP and p0^+^GFP by subcloning a Xho I - EcoRI restriction fragment to yield the plasmids p0^−^4BS^−^ and p0^+^4BS^−^. Homologous recombination between HSV-1 KOS and these plasmids yielded the HSV-1 recombinant viruses 0^−^4BS^−^ and 0^+^4BS^−^ (Table 1).
- ***p0^+^FLAG_24_*** **:** The plasmid p0^+^FLAG_24_ was created as follows. A plasmid, p0^+^GFP_24_, contained a Bgl II restriction site-*GFP* coding sequence-Spe I restriction site insertion between amino acids 23 and 24 of the ICP0 open-reading frame. A dsDNA oligonucleotide linker of the following sequence was synthesized, CCCAGATCTGGACTACAAGGACGATGACGACAAACTAGTCTG, digested with Bgl II and Spe I, and subcloned in place of the *GFP* sequence to create p0^+^FLAG_24_. The resulting gene encoded a FLAG-tagged protein that bore the amino acid sequence LDYKDDDDKL between amino acids 23 and 24 of ICP0. Homologous recombination between HSV-1 KOS and p0^+^FLAG_24_ yielded the HSV-1 recombinant virus 0^+^FLAG_24_ (Table 1).

### Construction of Recombinant HSV-1 Viruses

Infectious HSV-1 DNA was prepared by a protocol that relies upon dialysis to minimize shearing of genome-length HSV-1 DNA; this is a modification of a protocol that was generously provided by Karen Mossman (McMaster University, Hamilton, Ontario). Five 100 mm dishes of Vero cells (3×10^7^ cells) were inoculated with 5 pfu per cell of HSV-1 strain KOS. After 24 hours, cells were scraped, centrifuged, rinsed with PBS, resuspended in 7.0 ml of 200 mM EDTA pH 8.0, and transferred into a 15 ml conical. Proteinase K (75 µl of 10 mg/ml) and 375 µl of 10% SDS were added to virus-infected cells, and the tube was incubated in a rotisserie (hybridization) oven with slow rotation at 50°C for 16 hours. Proteins were removed by phenol : chloroform extraction, DNA was transferred into a 0.5–3.0 mL Slide-a-lyzer cassette (10,000 MW cutoff; Pierce Chemical Co., Rockford, IL), dialyzed against 0.1× standard saline citrate for 24 hours, aliquoted and frozen at −80°C until use.

Recombinant HSV-1 viruses were generated by co-transfecting the following DNA species into a 60 mm dish containing 8×10^5^ ICP0-complementing L7 cells: **1.** 1.8 µg infectious HSV-1 KOS DNA, **2.** 0.2 µg of an ICP0-expressiong plasmid (which promotes HSV-1 replication), and **3.** 1.0 µg of each ICP0^GFP^ reporter gene plasmid described above. Lipofectamine 2000 (Invitrogen Corporation) was used as the transfection reagent. After 12 hours, co-transfection medium was replaced with complete DMEM containing 1% methylcellulose and GFP^+^ plaques were selected on the stage of a TE2000 fluorescent microscope (Nikon Instruments, Lewisville, TX). GFP^+^ recombinant viruses were repeatedly passed in ICP0-complementing L7 cells until a uniform population of viruses was obtained that produced 100% GFP^+^ plaques, at which time Southern blot analysis was used to confirm that the anticipated *ICP0^GFP^* reporter gene was transferred into the HSV-1 recombinant virus.

### Western Blot Analysis

Vero cell cultures were established at a density of 3×10^5^ cells per well in 12-well plates, and were infected at an MOI of 5 pfu per cell. After 18 hours, proteins were harvested using mammalian protein extraction reagent (Pierce Chemical Co., Rockford, IL) supplemented with 1 M dithiothreitol and protease inhibitor cocktail set I (Calbiochem, La Jolla, CA). After heat denaturation, 20 µg of each protein sample and MagicMark™ XP protein MW markers (Invitrogen, Carlsbad, CA) were resolved in a 10% polyacrylamide gel with a 4% stacking gel, and were transferred to nitrocellulose membranes. Protein blots were blocked in phosphate-buffered saline (PBS) containing 5% nonfat dry milk, and were incubated overnight at 4°C in PBS +0.1% Tween-20 +5% nonfat dry milk containing a 1∶1000 dilution of primary antibodies. Specifically, the primary antibodies were either the mouse monoclonal 11060 antibody specific for a peptide between amino acids 20 to 105 of ICP0 (Santa Cruz Biotechnology, Santa Cruz, CA; Ref. [85]), mouse monoclonal H1083 antibody specific for a peptide between amino acids 395 to 775 of ICP0 (EastCoast Bio, North Berwick, MA; Ref. [86]), mouse monoclonal 58S antibody specific for a peptide between amino acids 700 to 1298 of ICP4 (Santa Cruz Biotechnology Clone 10F1; Ref. [87]), or a rabbit polyclonal anti-GFP antibody (Clontech Laboratories Inc.). Following incubation with primary antibodies, membranes were washed four times with PBS +0.1% Tween-20 (PBS-T), and were then incubated for 1 hour with secondary antibodies diluted 1∶20,000 in PBS +0.1% Tween-20 + nonfat 5% dry milk. The secondary antibodies were goat anti-rabbit IgG and goat anti-mouse IgG that were respectively conjugated to the infrared fluorescent dyes IRDye® 680 and IRDye® 800CW (LI-COR Bioscience, Lincoln, NE). Protein blots were washed three times in PBS-T, rinsed in PBS (to remove Tween-20), and were scanned for two-color fluorescence using the Odyssey Infrared imaging system (LI-COR Bioscience). Data were analyzed using Odyssey application software version 3.0.16 (LI-COR Bioscience).

### Northern Blot Analysis

Cultures of L7 or Vero cells were established at a density of 1.5×10^6^ cells per plate in 60 mm dishes, and inoculated with MOIs of 0.1 to 5 pfu per cell. RNA was isolated using Ultraspec RNA isolation reagent (Biotecx Inc., Houston, TX). Equal amounts of total RNA (10 µg) were electrophoretically separated on 1% formaldehyde agarose gels, blotted onto Zeta Probe GT nylon membranes (Biorad Laboratories, Hercules, CA), and hybridized with radiolabeled oligonucleotides specific for exon 3 of HSV-1 ICP0 mRNA (5′-ggagtcgctgatcactatggggtctctgttgtttgcaagg-3′), GFP mRNA (5′-atagacgttgtggctgttgtagttgtactccagcttgtgc-3′), HSV-1 glycoprotein D mRNA (5′-aggcccccagagacttgttgtaggagcattcggtgtactc-3′) or cellular GAPDH mRNA (5′-tgaccttggccaggggtgctaagcagttggtggtgcagga-3′). Oligonucleotides were end-labeled with [α-^32^P] dATP using terminal deoxynucleotidyl transferase (Promega Corporation, Madison, WI) and were hybridized to their target sequence via 16 hours of hybridization at 37°C in a solution containing 5 ng/ml labeled probe, 7% SDS, 120mM NaH_2_PO_4_, and 250mM NaCl. Excess probe was removed from membranes by sequential rinses in 0.1× standard saline citrate containing 0.1% SDS. Blots were exposed to phosphor screens, which were scanned and analyzed with a Cyclone PhosphorImager and OptiQuant software (Perkin Elmer, Boston, MA).

### Drug Treatments

In all experiments in which cycloheximide was used, 200 µM cycloheximide (Sigma Chemical Co., St. Louis, MO) was added to Vero cells 30 minutes prior to HSV-1 inoculation, and was maintained in cultures until the time of RNA harvest, or until the time at which protein translation was restored by removing cycloheximide, rinsing cultures twice, and thereafter incubating cells in complete DMEM containing 10 µg/ml actinomycin D (Sigma Chemical Co.).

### Immunofluorescent Staining of HSV-Infected Cells

The subcellular localization of FLAG-tagged ICP0 in HSV-1 0^+^FLAG_24_-infected cells was determined using an adaptation of a staining protocol that was generously provided by Roger Everett (MRC Virology Unit, Glasgow, Scotland). Cells were fixed with PBS containing 2% formaldehyde and 2% sucrose for 10 minutes and were permeabilized with 90% methanol for 10 minutes. HSV-1 Fc-γ receptors (glycoprotein E-I heterodimers; Ref. [88]) were blocked along with all other non-specific protein-binding sites by incubating fixed cells in a solution of PBS containing 0.5% fetal bovine serum and 10 µg/ml each of the γ-globulin fractions of human, donkey, and goat serum (PBS-F-Ig). Fixed and blocked cells were incubated for 16 hours with a 1∶1000 dilution of mouse FLAG-specific mouse monoclonal FLAG-M2 antibody (Sigma Chemical Co.). Excess antibody was removed by washing with PBS-F-Ig solution, and cells were incubated for 1 hour in 1∶1000 Alexa Fluor 594-conjugated goat α-mouse IgG secondary antibody (Molecular Probes, Eugene, OR). Excess secondary antibody was removed by washing with PBS-F-Ig solution, and cells were photographed at 20× magnification using a TE2000 inverted fluorescent microscope (Nikon Instruments, Lewisville, TX) and an Olympus DP72 digital camera (Olympus America Inc., Center Valley, PA).

### Co-Immunoprecipitation of FLAG-Tagged ICP0 and ICP4

Replicate 100 mm dishes containing 10^7^ Vero cells were inoculated with vehicle or 5 pfu per cell of HSV-1 KOS or HSV-1 0^+^FLAG_24_ in the presence of 200 µM cycloheximide. At 10 hours p.i., cycloheximide-containing medium was replaced with 10 µg/ml actinomycin D, and 6 hours was allowed for translation of accumulated IE mRNAs. At 16 hours p.i., 100 mm dishes of Vero cells were washed with ice-cold phosphate-buffered saline, and cells were lysed in either 0.5 ml of an NP40-based buffer or RIPA buffer. The NP40-based buffered contained 50 mM Tris (pH 7.4), 150 mM sodium chloride, 2 mM EDTA, 1% NP40, and 1× Halt protease inhibitor cocktail (Pierce Chemical Co.). RIPA buffer consisted of 50 mM Tris (pH 8.0), 150 mM sodium chloride, 1% NP40, 0.5% sodium deoxycholate, 0.1% SDS, and 1× Halt protease inhibitor cocktail. Cell lysates were incubated for 2 hours at 4°C on a rotisserie, cell debris was removed by centrifugation, and 10% of each lysate was reserved for Western blot comparison of relative levels of ICP0 and ICP4. Supernatants were pre-cleared by incubation with 1 µg normal mouse IgG and Protein A/G agarose beads (SantaCruz Biotechnology) for 30 min at 4°C. Pre-cleared supernatants were incubated with 15 µl of FLAG-M2 agarose beads (Sigma Chemical Company) overnight at 4°C. Immunocomplexes were washed four times with NP-40 buffer containing 500 mM NaCl or RIPA buffer, and were prepared for Western blot analysis by boiling in 2× Laemmli loading buffer prior to electrophoresis on an 8% polyacrylamide gel. Transfer and detection of blotted proteins on PVDF membranes was performed as described above.

## Supporting Information

Figure S1 Quantitative comparison of the accumulation of ICP0^−^GFP and ICP0^+^GFP protein accumulation following cycloheximide release. (A and B) Photomicrographs of Vero cells infected with 2.5 pfu per cell of (A) HSV-1 0^−^GFP or (B) HSV-1 0^+^GFP at 6, 12, and 18 hours p.i., as visualized by fluorescence microscopy (20× magnification). Vero cells were treated with vehicle or 200 ÂµM cycloheximide (CHX) from −0.5 to 6 hours p.i. (red box denotes CHX treatment), and cultures were released into medium containing no inhibitor from 6 to 18 hours p.i. (C) ICP0^GFP^ protein abundance between 3 and 18 hours p.i., as determined by flow cytometry. Each point represents the mean ± sem of ICP0^GFP^ abundance in n = 3 cultures x n = 25,000 cells per culture, where protein abundance was defined as the mean GFP fluorescence intensity scaled relative to background fluorescence, as defined by n = 12 uninfected cultures. The details of this methodology for quantifying GFP fluorescence are described in detail elsewhere [40].
(6.67 MB TIF)

Figure S2 Replicate blots of HSV-1 0^−^GFP and 0^+^GFP RNA samples shown in Figure 4 probed for (A) HSV-1 glycoprotein D mRNA or (B) cellular GAPDH mRNA, which encodes the glycolytic enzyme glyceraldehyde 3-phosphodehydrogenase. RNA was harvested from uninfected Vero cells (0 hours) or from cells between 3 and 12 hours after inoculation with 2.5 pfu per cell of HSV-1 0^−^GFP or HSV-1 0^+^GFP. Cells were treated with vehicle or 200 µM cycloheximide until the indicated time of total RNA harvest (10 µg per lane).
(7.21 MB TIF)
